# **“**Diagnose, Treat, and SUPPORT”. Clinical competencies in the management of older adults with aspiration pneumonia: a scoping review.

**DOI:** 10.1007/s41999-023-00898-4

**Published:** 2023-12-07

**Authors:** Yuki Yoshimatsu, Yoichi Ohtake, Mamiko Ukai, Taiju Miyagami, Toru Morikawa, Yoshinosuke Shimamura, Yuki Kataoka, Tadayuki Hashimoto

**Affiliations:** 1 Elderly Care, Queen Elizabeth Hospital, Lewisham and Greenwich NHS Trust, Stadium Rd, London, SE18 4QH UK; 2 Centre for Exercise Activity and Rehabilitation, School of Human Sciences, University of Greenwich, London, UK; 3 Scientific Research WorkS Peer Support Group (SRWS-PSG), Osaka, Japan; 4 Department of Internal Medicine, Imai Hospital, Hyogo, Japan; 5 Department of Family Medicine, Kameda Family Clinic, Tateyama, Japan; 6 Department of Health Data Science, Yokohama City University, Kanagawa, Japan; 7 Faculty of Medicine, Department of General Medicine, Juntendo University, Bunkyo City, Japan; 8 Department of General Medicine, Nara City Hospital, 1-50-1, Higashikideracho, Nara, 630-8305 Japan; 9 Department of Nephrology, Teine Keijinkai Medical Center, 1-40, Maeda 1-12, Teine, Sapporo, Hokkaido 006-8555 Japan; 10 Department of Internal Medicine, Kyoto Min-Iren Asukai Hospital, Tanaka Asukai-cho 89, Sakyo-ku, Kyoto, 606-8226 Japan; 11 Section of Clinical Epidemiology, Department of Community Medicine, Kyoto University Graduate School of Medicine, Yoshida Konoe-cho, Sakyo-ku, Kyoto, 606-8501 Japan; 12 Department of Healthcare Epidemiology, Graduate School of Medicine/Public Health, Kyoto University, Yoshida Konoe-cho, Sakyo-ku, Kyoto, 606-8501 Japan; 13 Department of General Medicine, Osaka Medical and Pharmaceutical University, Takatsuki, Japan

**Keywords:** Dysphagia, Pneumonitis, Pneumonia, Aspiration, Geriatric, Healthcare professional

## Abstract

**Background:**

Aspiration pneumonia in older adults is increasingly common, with a high care burden and morbidity. However, clinical competencies in its management have not been developed, and healthcare professionals struggle on how to care for these patients with multimodal treatment needs. Therefore, we conducted a scoping review to investigate what is known about the desired clinical competencies for the management of older adults with aspiration pneumonia, to utilise in clinical practice, education, and future research.

**Methods:**

First, we defined aspiration pneumonia according to a preliminary search. We then searched the literature on MEDLINE and CINAHL, focusing on studies involving patients aged 65 years old and older diagnosed with aspiration pneumonia. All settings were included, with the exception of intensive care units. Publication dates were limited to January 2011 to July 2022 and languages to English and Japanese. The extracted data were used to refine the preliminary competency framework developed by the Japan Aspiration pneumonia inter-Professional team Educational Program (JAPEP) in preparation of this study.

**Results:**

Ninety-nine studies were included. Following data extraction from these studies, 3 competencies were renamed, and 3 new competencies were added, to create a list of 12 competencies. These were Diagnosis, Treatment, Swallow Assessment, Underlying condition management, Nutrition, Oral management, Rehabilitation, Multidisciplinary team, Decision making, Prevention, Prognosis, and Palliative care.

**Conclusions:**

Our scoping review identified 12 clinical competencies required in the management of older adults with aspiration pneumonia, outlined in the phrase ‘Diagnose, Treat and SUPPORT’. We encourage healthcare professionals to share these competencies as a team to identify areas of unmet need and improve their patient care, with an emphasis on supportive care.

**Supplementary Information:**

The online version contains supplementary material available at 10.1007/s41999-023-00898-4.

## Key summary points

**Aim:**

We performed a scoping review to investigate the necessary clinical competencies for the management of AP in older adults, to serve as a baseline for multidisciplinary healthcare professionals to improve how they work with AP in the clinical, educational, and research settings.

**Findings:**

The clinical competencies for the management of aspiration pneumonia in older adults that were identified are summarized in the phrase ‘Diagnose, Treat, and SUPPORT’, where SUPPORT is an acronym for Swallow and nutrition intervention, Underlying condition management, Prognosis evaluation and decision making, Prevention and palliation, Oral management, Rehabilitation, and Team approach.

**Message:**

There is a particular demand for research and education in the area of supportive therapy for AP.

**Supplementary Information:**

The online version contains supplementary material available at 10.1007/s41999-023-00898-4.

## Introduction

Aspiration pneumonia (AP) is becoming an increasing concern for patients and clinicians in this unprecedented super-ageing society. AP in older adults is not only life-threatening, but also has a significant impact on the quality of life (QOL) of survivors and carers [1, 2]. However, the definition of AP remains variable among healthcare professionals [3]. Moreover, when managing AP in older patients, clinicians do not always consider the multimodal aspects of care that need to be provided, such as differential diagnosis [4], microbiology, eating, drinking, nutrition, swallowing assessment [5], cause assessment [6], oral hygiene and end-of-life issues [7, 8]. There is a need to address this variability in the management of patients with AP through a clinical and educational route in order to improve patient care.

To adequately train and guide healthcare professionals in the management of a complex condition such as AP in older adults, there is a need for a set framework of clinical competencies in this area. Clinical competencies (or professional competencies) are skills, knowledge and attributes that are valued specifically in the profession and setting. They are acquired abilities, and it is now mainstream in medical education to define them before developing the education curriculum [9]. They have been established in areas such as cancer and chronic disease [10–13]. However, despite being a disease of high incidence, morbidity, and complexity, and the subject of much research, clinical competencies have not been developed for the management of AP [3, 10, 11, 14, 15].

Therefore, given the clinically challenging and academically complicated characteristics of AP, this scoping review aimed to investigate the clinical competencies for the management of AP in older adults. We intend this study to serve as an initial exploratory baseline framework for multidisciplinary healthcare professionals in the multi-step process of improving how they work with AP in the clinical, educational, and research settings.

## Methods

This was a two-phase study. In the first phase, we defined AP through a search of literature. In the second phase, we performed a scoping review of literature to identify clinical competencies for the management of AP in older adults and used them to modify an original set of competencies that were developed through a simplified Delphi method.

### Phase 1: Definition of aspiration pneumonia

Despite the high prevalence of AP, there is still no clear unified definition or diagnostic criteria [3, 5, 16–19]. The variation in the definition of AP between institutions and geographical areas raises a technical issue in conducting a literature review on the topic. Therefore, to determine the common understanding of AP, we first performed a PubMed search for papers published in peer-reviewed journals in 2020–2021 with the term “aspiration pneumo*” in the title. This search was performed on 11 May 2022 and yielded 114 studies. We excluded case reports, grey literature (i.e.,: conference abstracts, expert opinions, letters) and those written in languages other than English or Japanese. For the remaining 65 studies, we performed a full-text screening. A further eight case reports were excluded, as were studies of postoperative or post-endoscopic AP studies, and studies that did not mention the diagnostic criteria for AP. Finally, 31 studies were included to determine the common definition of AP. Factors defining aspiration pneumonia were extracted from these articles. The results are shown in Table 1. Many studies chose their own set of criteria, while seven articles mentioned the use of previously reported criteria [8, 20–23]. From these studies, we found that AP was commonly defined as pneumonia occurring in the context of aspiration or dysphagia. In other words, we defined AP as “pneumonia in a patient with witnessed aspiration, frequent signs of aspiration, or risk factors for aspiration/dysphagia”.

**Table 1:** Factors used to define AP

Factor	Number of studies mentioning the factor
*Signs of pneumonia*	
Chest radiology	25
Symptoms of respiratory infection	19
Blood tests	11
*Aspiration/dysphagia*	
Risk factors of aspiration	16
Witnessed aspiration	12
Dysphagia	12
Clinically diagnosed dysphagia	5
Dysphagia diagnosed with FEES/VFSS	3
*Use of other guideline/criteria*	7

(*FEES* Flexible endoscopic evaluation of swallowing, *VFSS* videofluoroscopic swallowing study)

### Phase 2: Scoping review

We carried out the second phase of this study, the scoping review, in two stages. First, we developed a preliminary competency framework using a simplified Delphi method. Second, we conducted an evidence-based scoping review to expand the competency framework.

The preliminary competency framework was developed in the context of real clinical experience of the expert group, by members of the Japan Aspiration pneumonia inter-Professional team Educational Program (JAPEP), consisting of six general practitioners, a rehabilitation physician, respiratory physician, a psychosomatic physician, a dentist and a pharmacologist who were all clinically and academically experienced in the management of AP. The members developed a list of clinical competencies considered necessary in the management of older adults with AP in the common acute healthcare setting, using a simplified Delphi method. We chose the simplified Delphi method as this among other methods has been considered superior to more informal methods of expert opinion [24]. This initial list included diagnosis, treatment, swallow assessment, medication management, nutrition assessment, oral care, rehabilitation, multidisciplinary team, and ethics.

To further update these competencies, a scoping review was conducted to answer the question “What are the clinical competencies for the management of older adults with AP?”. We followed the JBI methodology for scoping reviews and the Preferred Reporting Items for Systematic Reviews and Meta-Analyses (PRISMA) extension for scoping reviews [25]. The PRISMA checklist for scoping reviews is shown in Supplementary 1. We registered the study protocol with the Open Science Framework (https://osf.io/ykbhf/) on 26 July 2022.

### Study participants

We defined study participants as patients aged 65 years and older with a diagnosis of AP. This included patients who had been diagnosed with AP in the past. We also included studies that focused on the care towards their caregivers and family members, and studies that surveyed healthcare professionals about their care of older patients with AP. Exclusion criteria included pneumonia in specific circumstances, such as postoperative pneumonia and postendoscopic pneumonia, pneumonia associated with endotracheal intubation or extubation, ventilator-associated pneumonia (VAP), hospital-acquired pneumonia (HAP) without mention of AP, studies of primary prevention of AP, and studies of investigations of AP that are not used in usual clinical practice (i.e., measurement of novel biomarkers or use of novel devices).

### Concept

We studied the clinical competencies desired of healthcare professionals who manage AP. We defined clinical competence as “the habitual and judicious use of communication, knowledge, technical skills, clinical reasoning, emotions, values, and reflection in daily practice for the benefit of the individual and community being served”, as suggested in previous literature [26]. In this context, competency could be skills aimed at assessing or treating pneumonia, improving prognosis, preventing further pneumonia, improving quality of life, or relieving symptoms. Competencies of multidisciplinary healthcare professionals (physicians, nurses, dieticians, pharmacologists, therapists, dentists, dental hygienists, social workers, etc.) were included. Skills focused solely on reducing health care costs were excluded. In addition to the nine competencies listed above, new competencies were added as needed.

### Context

Care provided in hospitals, clinics, nursing homes/care homes and community/home care were all included. There were no restrictions regarding cultural factors, geographical location, race or gender. Studies that focused on intensive care units were excluded.

### Types of sources

We included any original study published in a peer-reviewed journal. This included both experimental and quasi-experimental study designs, including randomised controlled trials, non-randomised controlled trials, before and after studies, and interrupted time series studies. In addition, analytical observational studies were considered for inclusion, including prospective and retrospective cohort studies, case–control studies and analytical cross-sectional studies. Qualitative studies, case reports, case series and review articles, text and opinion articles were excluded. Study languages were limited to English or Japanese to allow the authors to accurately interpret the content.

### Search strategy

The search strategy aimed to identify studies published in peer-reviewed journals. Unpublished or grey literature (conference abstracts and papers, letters, editorials, book chapters) were not included to maintain the scientific quality of the review. We searched PubMed and CINAHL on 10 July 2022. Studies published during 2011 to July 2022 were included, to best reflect the most recent clinical context, considering how the common understanding of what AP is and how to treat it has changed over the years [27]. The search formula is shown in Supplementary 2.

### Selection of studies

Following the search, we collated all identified citations and uploaded them into Rayyan (a web-tool for collaborative literature synthesis projects) [28] and removed duplicates. Following a pilot test, two independent reviewers (YY, MU, TM, and TM) blindly screened each title and abstract against the inclusion criteria. We retrieved the full text and citation details of potentially relevant sources. We also searched references from the following related guidelines: the Japanese Respiratory Society Adult Pneumonia Guidelines [20], The management and palliative care of pneumonia at home in end-stage dementia: a clinical guidance [29] and the Institute for Clinical Systems Improvement (ICSI) Health Care Guidelines "Palliative Care for Adults"[30]. We used citationchaser (an automated citation search tool) [31] to search for citations. The full texts of selected citations were assessed in detail against the inclusion criteria by two or more independent reviewers (YY, MU, TM, and TM). Reasons for exclusion of full-text evidence that did not meet the inclusion criteria were recorded. Disagreements between the reviewers at each stage of the selection process were resolved by two additional reviewers (TH and YO). The results of the search and the study inclusion process are presented using the PRISMA flowchart [32].

### Data extraction

Data were extracted independently by two blinded authors using a data extraction form. The data extraction form is shown in Supplementary 3 and was prepared according to the JBI Manual for Evidence Synthesis [33]. Any skill or knowledge related to the management of a patient with AP that was being discussed in the study as one of the main topics were considered as potential competencies. Disagreements were resolved through discussion and, if necessary, by involving two additional authors. Discussions involved raising reasons of why a certain skill should or should not be considered as a competency, by comparing the objectives of the study and clinical practice. Items collected were study characteristics, the clinical competencies discussed in the paper, the authors’ conclusions and any additional comments.

### Modifications from the protocol

Some modifications were made during the review process. First, we added post-intubation and post-extubation aspiration to the exclusion criteria. AP associated with intubation and extubation are similar to those associated with endoscopy procedures and are a different cohort from non-procedure related pneumonia. We added these exclusion criteria so that the results would focus on AP occurring in everyday settings. Secondly, an additional guideline was added to the selection of relevant guidelines of which the references were to be checked for relevant studies [29]. This was a new clinical guidance on the management of pneumonia in home care patients with end-stage dementia, developed in early 2022. Third, the data extraction items were changed. We had originally planned to extract competencies according to the ‘dimensions of professional competence’ as described by Epstein et al. [26]. However, through a data extraction trial, we found that it was not feasible or clinically relevant to categorise each clinical competency to a dimension of professional competence. The focus of this review was to enable clinicians to reflect on and improve their clinical practice and to guide their clinical teaching. Therefore, the data extraction was modified to collect competencies more familiar to clinicians (such as diagnosis, treatment, or prevention) rather than the more educational terms (such as cognitive, technical, or integrative).

## Results

### The study inclusion process

**Fig. 1:**
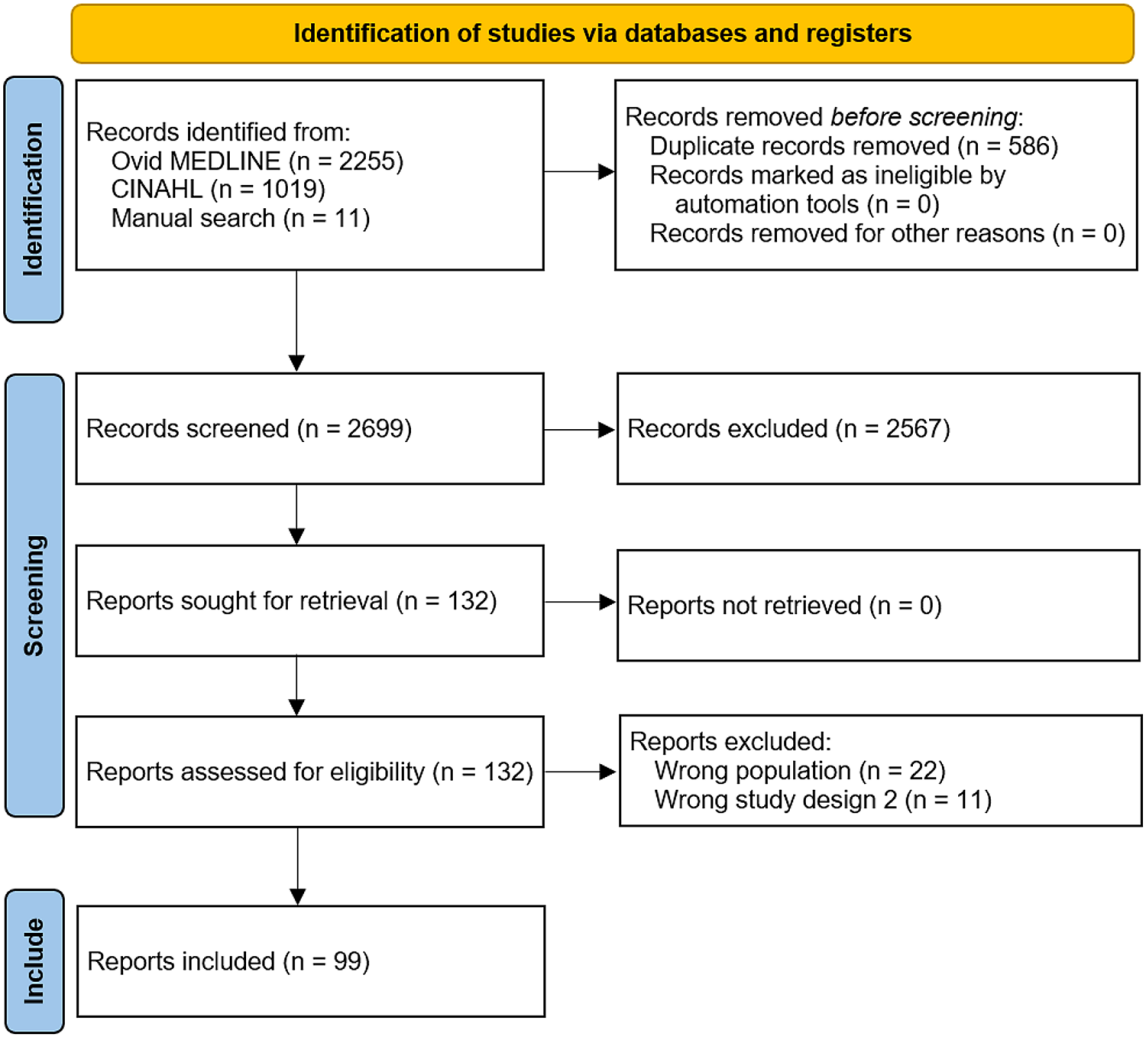
The study selection process. Through searching databases, 3274 reports were found, and an additional 11 studies were found through manual searches. After removing duplicates, 2699 reports were screened, of which 2567 were excluded. A total of 132 studies underwent full-text review, and 99 studies were included in the review

The process of study inclusion is shown in Fig. 1. After searching the databases, 3274 studies were identified, in addition to 11 studies from manual searches. After removing duplicates, 2699 studies remained and were uploaded for title and abstract screening, of which 2567 were excluded. The remaining 132 studies were screened for full text. Thirty-three studies were excluded for the following reasons: wrong population (*n* = 22) and wrong study design (*n* = 11). Ninety-nine studies were included in the final review. The full list of studies included and competencies discussed in each included study are presented in Supplementary 4.

Compared to the competences originally developed by our simplified Delphi method, some changes were identified. These changes are listed in Table 2. In the original version, ‘medication management’ meant the assessment and reduction/discontinuation of unnecessary medications taken regularly. As studies described not only the evaluation of medication but also of the underlying condition itself and non-medical treatments, this was changed to ‘underlying condition management’. Likewise, ‘oral care’ was changed to ‘oral management’ to include not only oral hygiene methods but also dental treatment. ‘Ethics’ has been changed to ‘decision making’ to better explain what is meant. In addition, three new competencies were identified and added to the list. These were prevention of recurrence, prognosis evaluation, and palliative care. There were no competencies which were not discussed in the included studies, so no competencies were omitted. In total, the updated list consisted of twelve competencies. Details of what was discussed under each competency are shown in Table 3. As some items may fall under multiple competencies, these were discussed among authors to decide which category was the most appropriate. Particularly, preventative measures such as rehabilitation, oral feeding methods, tube feeding techniques and oral care were categorised as the more specific categories of rehabilitation, swallow assessment, nutrition and oral management, respectively, rather than prevention.

**Table 2:** Clinical competencies in managing older adults with aspiration pneumonia

Original competencies from the simplified Delphi method	Updated competencies based on this review	Numbers of studies which discussed the competency
Diagnosis	(No change)	17
Treatment	(No change)	20
Swallow assessment	(No change)	22
Medication management	Underlying condition management	3
Nutrition	(No change)	21
Oral care	Oral management	5
Rehabilitation	(No change)	6
Multidisciplinary team	(No change)	4
Ethics	Decision making	3
(New)	Prevention	6
(New)	Prognosis	31
(New)	Palliative care	4

**Table 3:** The clinical competencies in managing older adults with aspiration

Competency	Content
Diagnosis	Knowing the characteristics of AP and differences from CAP, HAP, HCAP, NHCAP, aspiration pneumonitis Detecting pathogens, assessing risk of resistant bacteria Evaluating X-ray and CT images Diagnosing the underlying condition causing the dysphagia or aspiration Recording and coding correctly on medical records
Treatment	Appropriate antibiotic treatment Fluid and electrolyte management Airway and breathing, and sputum management
Swallow assessment	Early consultation to a speech therapist Observation of eating and drinking habits Assessment with videofluoroscopy, videoendoscopy Assessment of silent aspiration and cough effectiveness Appropriate diet and liquid modification and feeding techniques
Underlying condition management	Management of dysphagia and aspiration related with the underlying condition Drug review
Nutrition	Early initiation of oral intake (avoiding unnecessary fasting) Modification of diet and liquids according to swallow function Evaluation of nutritional status and intake Supplementation of inadequate oral intake Appropriate usage of tube feeding and prevention of complications
Oral management	Evaluation of oral status (OHAT, ROAG) Frequent oral care Availability of guidance and resources for oral care on the ward Appropriate use and cleaning of dentures Dental treatment
Rehabilitation	Early initiation of physiotherapy and swallow training Maintenance of daily activities Utilisation of devices (TESS)
Multidisciplinary team	Discussions regarding difficult decision making Developing protocols for AP management, risk feeding Comprehensive team intervention Providing efficient training for healthcare professionals
Decision making	Acknowledging the complexity of decision making Decisions on administering, withholding, or withdrawing antibiotics, hydration, risk feeding, hospitalisation, and resuscitation Accepting AP as a natural course of decline in terminal cases Understanding the ethical challenges and dilemmas of difficult decision making
Prevention	Oral Care Vaccination Drug review (discontinuation of risky drugs, appropriate use of preventative drugs)
Prognosis	Awareness of poor prognosis compared to non-AP Evaluation of severity, prognosis and survival rate utilisation of prognostic factors Communicating the perceived prognosis to patients and families
Palliative care	Subjective and objective symptom assessments Symptom control (Discomfort, pain, dyspnoea, cough, rattling breath)

*AP* aspiration pneumonia, *CAP* community acquired pneumonia, *HAP* hospital acquired pneumonia, *HCAP* healthcare-associated pneumonia, *NHCAP* nursing and healthcare-associated pneumonia, *CT* computed tomography, *OHAT* oral health assessment tool, *ROAG* Revised Oral Assessment Guide, *TESS* transcutaneous electrical sensory stimulation

The frequency and extent to which each competency was discussed in the studies varied widely, as shown in Table 2. Diagnosis, treatment, swallow assessment, nutrition and prognosis were discussed in 15 or more studies, whereas underlying condition management, oral management, rehabilitation, multidisciplinary team, decision making, prevention and palliative care were mentioned in only a few studies.

**Fig. 2:**
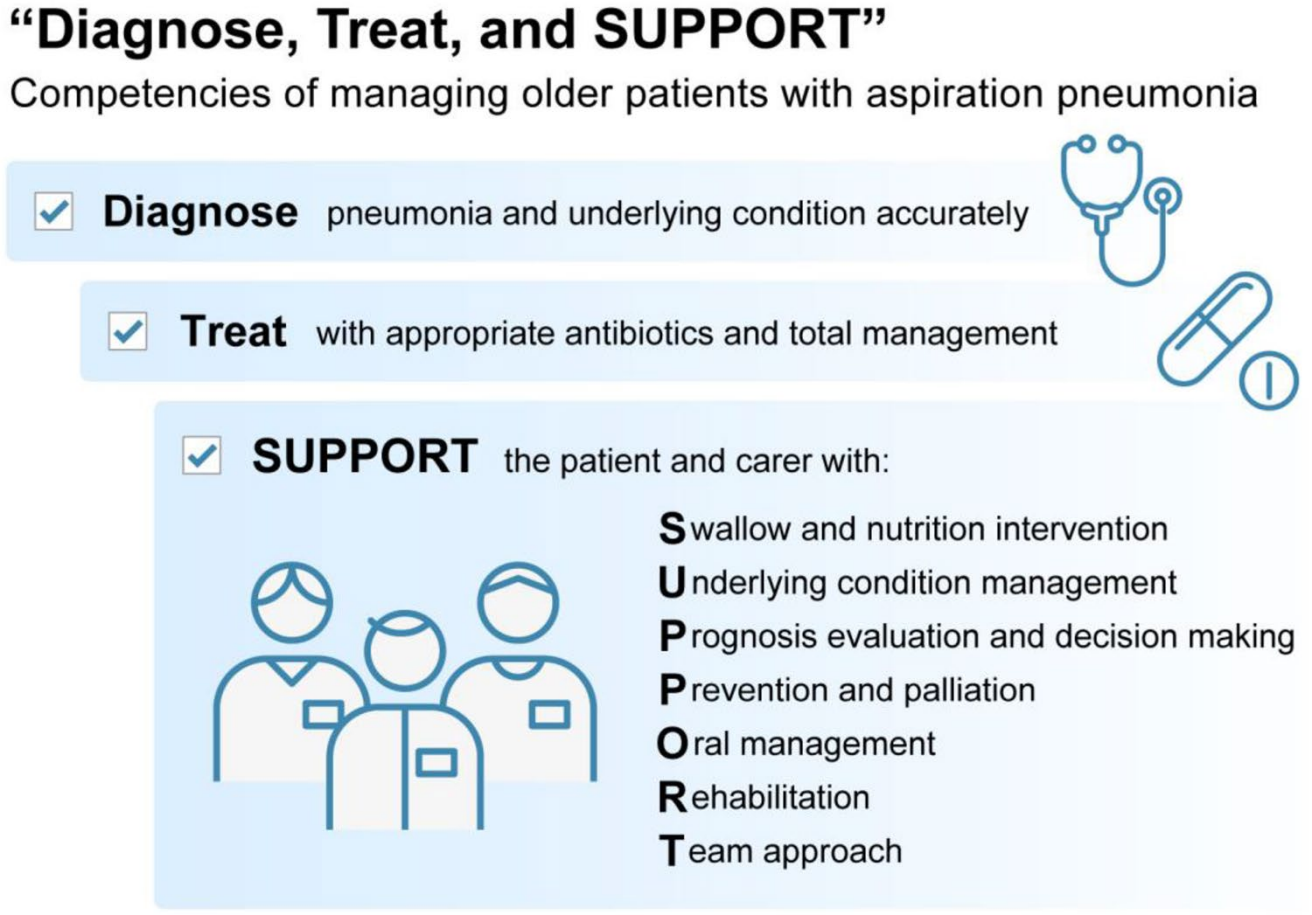
Clinical competencies in the management of older adults with aspiration pneumonia. The clinical competencies required in the management of older patients with aspiration pneumonia can be summarised by the phrase ‘Diagnose, Treat and SUPPORT’. The first key steps are to accurately diagnose the pneumonia and underlying conditions and to treat with appropriate antibiotics. The next important steps are to SUPPORT the patient and carer as a multidisciplinary team according to the modalities shown

Using the 12 clinical competencies extracted from the studies, we created a key phrase to describe the competencies in the management of older adults with AP: ‘Diagnose, Treat and SUPPORT’ (Fig. 2). This phrase brings together the twelve competencies, where ‘Diagnose’ stands for diagnosis, ‘Treat’ for treatment, and SUPPORT is an acronym of the remaining ten competencies, as shown in Fig. 2. The phrase also effectively conveys the importance that the first key steps of suspected AP management are to accurately diagnose the pneumonia and underlying conditions and to treat with appropriate antibiotics. The next important steps are to SUPPORT the patient and carer as a multidisciplinary team according to the modalities such as swallow assessment, underlying condition management, and so on.

## Discussion

We conducted a scoping review of the literature on clinical competencies in the management of older adults with AP. The final set of twelve competencies were integrated into the phrase ‘Diagnose, Treat, and SUPPORT’ to be used in the clinical and educational settings (Fig. 2). While there are many studies that focus on a particular dimension of care for AP, to our knowledge this was the first study to integrate them to visualise what is needed as a whole.

### ‘Diagnose, Treat, and SUPPORT’

The phrase ‘Diagnose, Treat, and SUPPORT’ outlines the clinical competencies required in the management of older adults with aspiration pneumonia. It is intended to be shared with team members to identify areas that need to be addressed and to ensure the necessary multidimensional care. The phrase shows that the first essential step is to accurately diagnose the pneumonia and underlying conditions. This includes differentiating other conditions [4, 6, 15], microbiological testing, assessing severity and evaluating any underlying conditions that may have put the patient at risk of AP [6, 15]. The second key step is to treat with appropriate antibiotics and manage their overall care with adequate fluids, electrolyte correction, oxygen and airway management, secretion management, etc. These two critical steps are usually led by the physician and are an absolute necessity in the management of patients with AP. As the optimal choice and length of antibiotic treatment are still controversial, the appropriate treatment should be discussed depending on the local guidelines and individual patient condition [7, 20, 27, 34, 35]. The next important steps are to SUPPORT the patient and carer as a multidisciplinary team according to the modalities as shown in the mnemonic acronym: Swallow and nutrition intervention, Underlying condition management, Prognosis evaluation and decision making, Prevention and palliation, Oral management, Rehabilitation, all conducted with a Team approach. This phrase ‘Diagnose, Treat, and SUPPORT’ is intended to help healthcare professionals remember what needs to be done in everyday clinical practice.

### Comparison to other studies

There are no studies that have identified the competencies needed in the management of patients with AP. In other areas of medicine, such as chronic disease and its prevention, core competencies were developed by the National Association of Chronic Disease Directors (NACDD) in 2007 and have since been updated to reflect recent changes [12]. Paediatric care [10] and cancer [11, 13] are also areas where some sets of competencies have been developed. These competencies appear to be more focused on education and management, including domains such as ‘design and update programs’, ‘manage people’, ‘lead strategically’, under which more detailed content is presented. We aimed our list of competencies at healthcare professionals working at the bedside and in the community. We focused on using terms and themes that would be directly relevant to clinicians, such as ‘palliation’, ‘decision making’, and ‘team approach’. For this reason, we modified our protocol so as not to use the dimensions that have been introduced in a more educational setting (as mentioned in the methods sections) [26], and the reason for developing the phrase ‘Diagnose, Treat, and SUPPORT’. As healthcare professionals become accustomed to these competencies and bedside clinical practice improves, the next step may be to consider the benefits of developing more education and management focused competencies in AP.

### Implications of this study

This review highlights a number of clinically and academically important implications. Clinical implications include use in daily clinical practice to identify areas of unmet need and improve patient care. Until now, while there have been guidance on the management of dysphagia or individual underlying conditions such as stroke, there has been no comprehensive guidance for the management of AP. Healthcare professionals from all disciplines can use the list of competencies to identify and fulfill their role as a member of a multidisciplinary team. The competencies would also be useful as a guide for clinical teaching and for education and training outside of the clinical setting. In addition, they could form the basis for changes in health policies and systems.

The review also identified areas for further research. In particular, more studies are needed on the supportive care competencies represented by the mnemonic acronym ‘SUPPORT’, especially underlying condition management, oral management, rehabilitation, multidisciplinary team, decision making, prevention and palliative care. Although studies have been published on these individual topics, there are few studies conducted in the context of AP. There is also a need to determine how to train and assess healthcare professionals in these competencies. Our study will hopefully provide a basis for further development, with future refinements and regular review and updating.

### Limitations

There are some limitations to this study, the first of which is that we did not use a Delphi method to decide whether new competencies should be added to the original set of competencies. Additionally, we settled for a simplified Delphi method in developing the initial set of competencies. However, we held several discussions among the multidisciplinary members of JAPEP, who represent a common team in the management of AP in Japan, to minimise the effect of this limitation. Assessing the completeness and representativeness of the list is a necessary future step, as is also to involve a speech therapist and geriatrician in the team, as we implement the framework. For example, comprehensive geriatric assessment (CGA) and frailty assessments are expected to be important aspects in the management of AP in older adults. Other limitations include the language of the searched studies and the year of publication. However, 99 reports were selected from an initial number of 3274. This relatively large number is likely to reflect the trends in current practice and research. We recognise that competencies can be interpreted in different ways depending on how a paper is read and understood. For example, two people reading the same paper could identify different competencies from that study. This is why we designated two independent reviewers for each step of the process from screening to data extraction, supported by two additional reviewers to resolve any discrepancies.

## Conclusion

Our scoping review identified 12 clinical competencies required in the management of older adults with aspiration pneumonia, outlined in the phrase ‘Diagnose, Treat and SUPPORT’. There is a particular demand for research and education in the area of supportive therapy for AP, as expressed in the acronym SUPPORT. We encourage healthcare professionals to share these competencies as a team to identify areas of unmet need and improve their patient care. Methods to effectively train and assess healthcare professionals in these competencies are awaited.

### Supplementary Information

Below is the link to the electronic supplementary material.

Supplementary file1 (PDF 150 KB)

Supplementary file2 (PDF 124 KB)

Supplementary file3 (PDF 105 KB)

## Data Availability

All data are applicable in the paper.
