# Supplementary material for: “Diagnose, Treat, and SUPPORT”. Clinical competencies in the management of older adults with aspiration pneumonia: a scoping review
Source: Eur Geriatr Med. 2023 Dec 7;15(1):57–66. doi: 10.1007/s41999-023-00898-4 (PMC10876713; doi:10.1007/s41999-023-00898-4)
Supplement: Supplementary file 2 — Supplementary file2 (PDF 124 KB) [file 41999_2023_898_MOESM2_ESM.pdf]

**Title:** " **Diagnose, Treat, and SUPPORT". Clinical competencies in the management of older adults with aspiration pneumonia: a scoping review.**

**Journal:** European Geriatric Medicine

**Authors:** Yuki Yoshimatsu[1,2,3] Yoichi Ohtake[4], Mamiko Ukai[5,6], Taiju Miyagami[7], Toru Morikawa, Yoshinosuke Shimamura[3,9] Yuki Kataoka[3,10,11,12] Tadayuki Hashimoto[13]

**Author affiliations:**

1. Elderly Care, Queen Elizabeth Hospital, Lewisham and Greenwich NHS Trust, London, UK
2. Centre for Exercise Activity and Rehabilitation, School of Human Sciences, University of Greenwich, London, UK
3. Scientific Research WorkS Peer Support Group (SRWS-PSG), Osaka, Japan
4. Department of internal medicine, Imai Hospital, Hyogo, Japan
5. Department of Family Medicine, Kameda Family Clinic Tateyama, Japan.
6. Department of Health Data Science, Yokohama City University, Kanagawa, Japan.
7. Department of General Medicine, Juntendo University Faculty of Medicine, Japan
8. Department of General Medicine, Nara City Hospital, 1-50-1, Higashikideracho, Nara, 630-8305, Japan
9. Department of Nephrology, Teine Keijinkai Medical Center, 1-40, Maeda 1-12, Teine, Sapporo, Hokkaido, 006-8555, Japan
10. Department of Internal Medicine, Kyoto Min-Iren Asukai Hospital, Tanaka Asukai-cho 89, Sakyo-ku, Kyoto 606-8226, JAPAN
11. Section of Clinical Epidemiology, Department of Community Medicine, Kyoto University Graduate School of Medicine, Yoshida Konoe-cho, Sakyo-ku, Kyoto 606-8501, JAPAN
12. Department of Healthcare Epidemiology, Kyoto University Graduate School of Medicine / Public Health, Yoshida Konoe-cho, Sakyo-ku, Kyoto 606-8501, JAPAN
13. Department of General Medicine, Osaka Medical and Pharmaceutical University, Takatsuki, Japan

**Corresponding author:** Yuki Yoshimatsu, MD, PhD, Master of Swallowing Disorders

E-mail: yukitsukihana0105@gmail.com

**Supplementary 2. Search formula**

A. Search formula for PubMed

| | | |
|---|---|---|
| 1 | exp Pneumonia, Aspiration/ | 6266 |
| 2 | exp Respiratory Aspiration/ | 1567 |
| 3 | aspiration pneumoni*.mp. | 5268 |
| 4 | 1 or 2 or 3 | 11200 |
| 5 | exp Professional Competence/ | 126823 |
| 6 | exp "Quality of Health Care"/ | 7839111 |
| 7 | exp Patient Care Management/ | 881644 |
| 8 | exp Therapeutics/ | 5011814 |
| 9 | exp Nursing/ | 261145 |
| 10 | PC.fs. | 1418675 |
| 11 | exp Rehabilitation/ | 340877 |
| 12 | exp Terminal Care/ | 55645 |
| 13 | exp Health Communication/ | 3056 |
| 14 | exp Clinical Decision-Making/ | 14461 |
| 15 | Competenc*.mp. | 217845 |
| 16 | Quality.mp. | 1430284 |
| 17 | management.mp. | 1497544 |
| 18 | Therap*.mp. | 6884013 |
| 19 | Nurs*.mp. | 786926 |
| 20 | prevention.mp. | 1837420 |
| 21 | Rehabilitation.mp. | 351849 |
| 22 | Care.mp. | 2441882 |
| 23 | Communication*.mp. | 437388 |
| 24 | Decision.mp. | 421469 |
| 25 | reasoning.mp. | 26908 |
| 26 | or/5-25 | 16196077 |
| 27 | 4 and 26 | 8679 |

28	(child* or stepchild* or step-child* or kid or kids or girl or girls or boy or boys or teen* or youth* or youngster* or adolescent* or adolescence or preschool* or pre-school* or kindergarten* or school* or juvenile* or minors or p?ediatric* or PICU).ti,ab. or exp child/	3115177

29	case reports.pt.	2278560

30	exp animals/ not humans.sh.	5025514

31	28 or 29 or 30	9952021

32	27 not 31	5317

33	limit 32 to yr="2011 -Current"	2255

B. Search formula for CINAHL

S1	(MH "Pneumonia, Aspiration")	1,739

S2	(TI "aspiration pneumoni*" OR AB "aspiration pneumoni*")	1,484

S3	S1 OR S2	2,605

S4	(MH "Professional Competence"+)	700

S5	(MH "Quality of Health Care"+)	6,922

S6	(MH "Patient Care"+)	5,206

S7	(MH Therapeutics+)	1,684,825

S8	(MH "Safety (Iowa NIC)+")	39

S9	(MH Rehabilitation+)	314,457

S10	(MH "Terminal Care"+)	3,662

S11	(MH "Decision Making, Clinical"+)	4,411

S12	(TI Competenc* OR AB Competenc*) OR (TI Quality OR AB Quality) OR (TI management OR AB management) OR (TI Therap* OR AB Therap*) OR (TI Nurs* OR AB Nurs*) OR (TI prevention OR AB prevention) OR (TI Rehabilitation OR AB Rehabilitation) OR (TI Care OR AB Care) OR (TI Communication* OR AB Communication*) OR (TI Decision OR AB Decision) OR (TI reasoning OR AB reasoning)	2,664,908

S13	S4 OR S5 OR S6 OR S7 OR S8 OR S9 OR S10 OR S11 OR S12	3,566,445

S14	S3 AND S13	1,738

S15	((TI child* OR AB child*) OR (TI stepchild* OR AB stepchild*) OR (TI step-child* OR AB step-child*) OR (TI kid OR AB kid) OR (TI kids OR AB kids) OR (TI girl OR AB girl) OR (TI girls OR AB girls) OR (TI boy OR AB boy) OR (TI boys OR AB

boys) OR (TI teen* OR AB teen*) OR (TI youth* OR AB youth*) OR (TI youngster* OR AB youngster*) OR (TI adolescent* OR AB adolescent*) OR (TI adolescence OR AB adolescence) OR (TI preschool* OR AB preschool*) OR (TI pre-school* OR AB pre-school*) OR (TI kindergarten* OR AB kindergarten*) OR (TI school* OR AB school*) OR (TI juvenile* OR AB juvenile*) OR (TI minors OR AB minors) OR (TI pediatric* OR AB pediatric*) OR (TI paediatric* OR AB paediatric*) OR (TI PICU OR AB PICU)) OR (MH child+) 8,360

S16 (MH animals+) NOT (MH human) 91,982

S17 S15 OR S16 91,997

S18 S14 NOT S17 1,726

S19 limit S18 from 20110101 to 20221231
