# Supplementary material for: “Diagnose, Treat, and SUPPORT”. Clinical competencies in the management of older adults with aspiration pneumonia: a scoping review
Source: Eur Geriatr Med. 2023 Dec 7;15(1):57–66. doi: 10.1007/s41999-023-00898-4 (PMC10876713; doi:10.1007/s41999-023-00898-4)
Supplement: Supplementary file 3 — Supplementary file3 (PDF 105 KB) [file 41999_2023_898_MOESM3_ESM.pdf]

Title: “ Diagnose, Treat, and SUPPORT”. Clinical competencies in the management of older adults with aspiration pneumonia: a scoping review.

Journal: European Geriatric Medicine

Authors: Yuki Yoshimatsu[1,2,3] Yoichi Ohtake[4], Mamiko Ukai[5,6], Taiju Miyagami[7], Toru Morikawa, Yoshinosuke Shimamura[3,9] Yuki Kataoka[3,10,11,12] Tadayuki Hashimoto[13]

Author affiliations:

1. Elderly Care, Queen Elizabeth Hospital, Lewisham and Greenwich NHS Trust, London, UK
2. Centre for Exercise Activity and Rehabilitation, School of Human Sciences, University of Greenwich, London, UK
3. Scientific Research WorkS Peer Support Group (SRWS-PSG), Osaka, Japan
4. Department of internal medicine, Imai Hospital, Hyogo, Japan
5. Department of Family Medicine, Kameda Family Clinic Tateyama, Japan.
6. Department of Health Data Science, Yokohama City University, Kanagawa, Japan.
7. Department of General Medicine, Juntendo University Faculty of Medicine, Japan
8. Department of General Medicine, Nara City Hospital, 1-50-1, Higashikideracho, Nara, 630-8305, Japan
9. Department of Nephrology, Teine Keijinkai Medical Center, 1-40, Maeda 1-12, Teine, Sapporo, Hokkaido, 006-8555, Japan
10. Department of Internal Medicine, Kyoto Min-Iren Asukai Hospital, Tanaka Asukai-cho 89, Sakyo-ku, Kyoto 606-8226, JAPAN
11. Section of Clinical Epidemiology, Department of Community Medicine, Kyoto University Graduate School of Medicine, Yoshida Konoe-cho, Sakyo-ku, Kyoto 606-8501, JAPAN
12. Department of Healthcare Epidemiology, Kyoto University Graduate School of Medicine / Public Health, Yoshida Konoe-cho, Sakyo-ku, Kyoto 606-8501, JAPAN
13. Department of General Medicine, Osaka Medical and Pharmaceutical University, Takatsuki, Japan

Corresponding author: Yuki Yoshimatsu, MD, PhD, Master of Swallowing Disorders

E-mail: yukitsukihana0105@gmail.com

Supplementary 3. Data extraction form

**Item**	**Answer**
Title	
Author	
Year	
Design	
Professional competence discussed in the paper (select all that apply, specify competency if other)	Diagnosis, Treatment, Swallow assessment, Medication management, Nutrition, Oral care, Rehabilitation, Multidisciplinary team, Ethics, Other (___________)
Competence in detail	
Study summary	
Author’s conclusions	
Notes	
